# Stress-related emotional and behavioural impact following the first COVID-19 outbreak peak

**DOI:** 10.1038/s41380-021-01219-6

**Published:** 2021-08-04

**Authors:** Asaf Benjamin, Yael Kuperman, Noa Eren, Ron Rotkopf, Maya Amitai, Hagai Rossman, Smadar Shilo, Tomer Meir, Ayya Keshet, Orit Nuttman-Shwartz, Eran Segal, Alon Chen

**Affiliations:** 1 The Ruhman Family Laboratory for Research on the Neurobiology of Stress, Department of Neurobiology, Weizmann Institute of Science, Rehovot, Israel; 2 Department of Stress Neurobiology and Neurogenetics, Max-Planck Institute of Psychiatry, Munich, Germany; 3 Department of Veterinary Resources, Weizmann Institute of Science, Rehovot, Israel; 4 Department of Life Sciences Core Facilities, Weizmann Institute of Science, Rehovot, Israel; 5 Department of Psychological Medicine, Schneider Children’s Medical Center of Israel, Petach Tikva, Israel; 6 Sackler Faculty of Medicine, Tel Aviv University, Tel Aviv, Israel; 7 Department of Computer Science and Applied Mathematics, Weizmann Institute of Science, Rehovot, Israel; 8 School of Social Work, Sapir College, D.N. Hof Ashkelon, Israel

**Keywords:** Neuroscience, Psychiatric disorders

## Abstract

The COVID-19 pandemic poses multiple psychologically stressful challenges and is associated with an increased risk for mental illness. Previous studies have focused on the psychopathological symptoms associated with the outbreak peak. Here, we examined the behavioural and mental-health impact of the pandemic in Israel using an online survey, during the six weeks encompassing the end of the first outbreak and the beginning of the second. We used clinically validated instruments to assess anxiety- and depression-related emotional distress, symptoms, and coping strategies, as well as questions designed to specifically assess COVID-19-related concerns. Higher emotional burden was associated with being female, younger, unemployed, living in high socioeconomic status localities, having prior medical conditions, encountering more people, and experiencing physiological symptoms. Our findings highlight the environmental context and its importance in understanding individual ability to cope with the long-term stressful challenges of the pandemic.

## Introduction

The severe acute respiratory syndrome coronavirus 2 (SARS-CoV-2), which causes the coronavirus disease 2019 (COVID-19), has infected and taken the lives of millions of people around the world [1]. The pandemic has dramatically affected virtually all aspects of our lives: It has led to the largest global recession since the Great Depression [2], and to extreme social isolation due to changes in educational and work activities, local lockdowns, and international travel restrictions. Social isolation and financial instability, together with a fear of contracting COVID-19 and uncertainty of the future, pose substantial psychological stressors for the general population [3]. It is likely that the pandemic induces a considerable degree of fear, worry and concern in the general population.

Studies examining the impact of this pandemic on people’s mental health, and ways of mitigating adverse mental-health consequences for vulnerable subgroups, are critically needed. So far, most of the work in this area has addressed the acute mental-health impact of the COVID-19 pandemic, measured during the outbreak peak. Indeed, despite geographical and cultural differences, several studies have provided largely consistent results in these aspects: A study  of Chinese residents found that 54% of 1,210 respondents rated the psychological impact of the COVID-19 outbreak as moderate or severe [4]. In an Australian study using an online survey during the outbreak peak, 78% of 5,070 respondents reported worsening of their mental health since the outbreak [5]. An additional study performed on a Spanish cohort (*n* = 3,840), found that age, economic stability, and being male, were all negatively correlated with reports of depression, anxiety, and PTSD during the initial stage of the COVID-19 pandemic [6]. These and other studies describe the anxiety induced by the pandemic and its negative correlation with sleep quality and social support, both in the general population and in susceptible groups, such as healthcare staff members [7–9] and self-isolated people [10, 11]. It was also shown that individuals across western and northern Europe have responded in psychologically similar ways despite the differences in government approaches to the pandemic [12].

To further shed light on this emerging global picture, we set out to assess the long-term mental health-related effects of the pandemic on the adult population in Israel using an online survey that could be filled once a day. During the six weeks between April 28th and June 9th 2020, we collected 12,125 responses from 4,933 adult respondents (see ‘Research sample’ description in the ‘Methods’ section). The respondents agreed to answer a two-stage online questionnaire, where in the first stage they reported on COVID-19-related physiological symptoms and behaviours together with background demographic and medical information, and in the second stage on the effects of COVID-19 on their psychological and emotional state (see ‘Methods’ section). Importantly, our data were collected after the first outbreak peak, and thus reflect a period in which people have had the chance to adapt to the new circumstances, rather than the initial reactions to the outbreak. These six weeks of data collection allowed us a broad and dynamic view of the period between the first and the second outbreaks.

## Results

### COVID-19-linked stressors induced mainly non-self-centred concerns

**Fig. 1:**
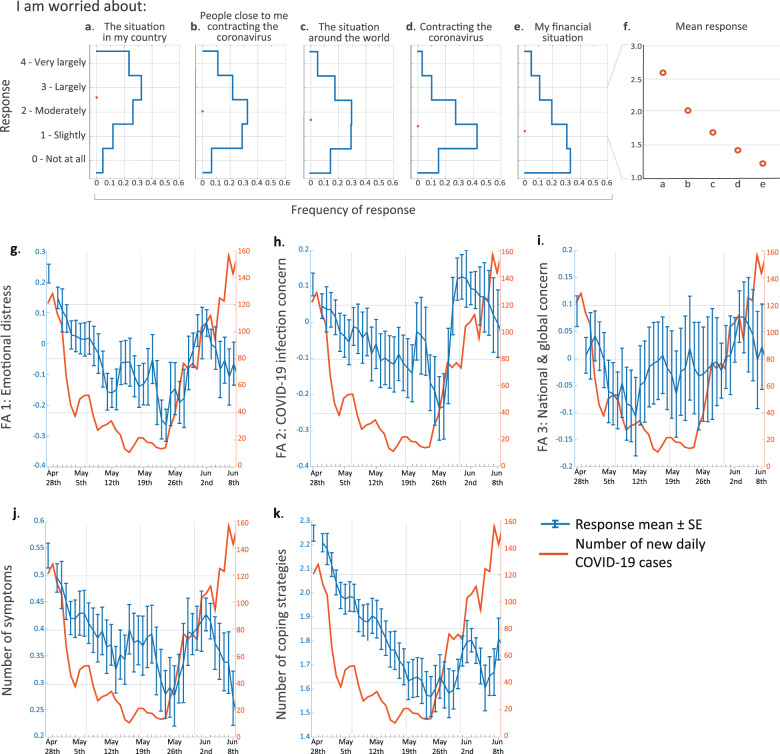
Concerns induced by COVID-19 and their temporal dynamics. **a**–**e** Blue lines represent distributions of participants’ first responses for specific reasons for worry among all respondents. Orange circles represent response means. **f** Zoomed-in view of the response means shown in **a**–**e**. Note that all five SE ranges are shorter than the circle diameters and were thus omitted from the plot. **g**–**k** Daily means and SE of **g** emotional distress, **h** COVID-19 infection concern, **i** national and global concern, **j** number of stress-related symptoms and **k** number of stress-coping strategies. Orange curves indicate the number of newly diagnosed COVID-19 patients as published by the Israeli Ministry of Health (www.health.gov.il). Kendall’s correlation coefficient with Bonferroni correction for multiple comparisons: **g** Tau = 0.03; *p* = 0.009; **h** Tau = 0.01; *p* = 0.733; **i** Tau = −0.01; *p* = 0.963; **j** Tau = 0.03; *p* = 0.0186; **k** Tau = 0.11; *p* < 1e−21.

While recent studies have begun to address the psychological and emotional effects of the pandemic, less is known regarding the underlying motives. Here, we examine the specific reasons that may underlie the psychological and emotional effects of the pandemic. Thus, to try and assess what people are most worried about during the pandemic, we asked respondents to rate the extent to which they were concerned about specific issues. Despite the many unknowns at the time about the COVID-19 disease and its potential effects on our personal lives in the future, respondents reported lower levels of concern about their personal situation - namely, contracting the coronavirus and their financial state - than they did about the situation in their country and about people close to them contracting the virus (Fig. 1a–f; Friedman’s test with post hoc Bonferroni correction for multiple comparisons: *n* = 4882; χ^2^ = 4971; df = 4; for all pairwise comparisons: *p* < 1e−8).

In addition to specific reasons for worry, we looked into reports of distress-related symptoms and emotions more generally, using questions from the anxiety and depression subscales of the brief symptom inventory 18 (BSI-18 [13]) and from the perceived stress scale (PSS [14]) (Supplementary Fig. 1). In our cohort, the mean BSI-18 scores for anxiety and depression were 0.72 ± 0.8 and 0.42 ± 0.61, respectively. These scores are similar to the Israeli norm (anxiety: 0.85 ± 0.71; depression: 0.7 ± 0.69), which is based on a nationwide representative sample of 510 community respondents between the ages of 35 and 65 years [15]. We further asked about experiencing several stress-related physiological symptoms and about the stress-coping strategies used (part of the brief-COPE questionnaire [16]). To gain a more integrative and concise description of respondents’ emotional responses, we examined to what extent answers were correlated across questions and could thus be compactly represented by a smaller number of factors. We used factor analysis on the answers to the distress- and worry-related questions, which revealed three principal factors or components (see ‘Methods’ section and Supplementary Fig. 2). The three factors respectively corresponded to questions related to (1) general emotional distress and worrying about your financial situation (henceforth: ‘general emotional distress’); (2) worrying about contracting COVID-19 and about people close to you contracting COVID-19 (henceforth: ‘COVID-19 infection concern‘), and (3) worrying about the situation in Israel and around the world (henceforth: ‘national and global concern’). In most of the following analyses, we focus on these three measures, along with the number of stress-related physiological symptoms experienced and the number of stress-coping strategies used.

### Reported distress correlates with the number of new COVID-19 cases

Next, we examined whether and how responses changed over time. We observed qualitatively similar characteristics between the temporal dynamics of our five main scores and the number of new daily COVID-19 cases as published by the Israeli Ministry of Health (health.gov.il; Fig. 1g–k). All of these scores seem to gradually decline over the first several weeks, together with the decline in the numbers of new daily COVID-19 cases. Similarly, around May 26th, as the numbers of new cases started to rise, so did the reported scores. However, about a week later, despite the continued rise in new cases, these scores started to decline, presumably reflecting an adaptation or behavioural habituation to this new situation. A study that assessed mental health in different societies found that the total number of cases is correlated with anxious thoughts about COVID-19 [17]. Here we add that the COVID-19 concern parameter is sensitive to the change in cases, not just the absolute number. Notably, although the dynamics of the national-global concern seem somewhat similar to those of the other scales (a decrease followed by an increase and another decrease), its initial rise starts earlier and it seems largely more stable compared to the other scales (Fig. 1i).

To rigorously quantify these relationships, we constructed mixed-effects models for all the collected responses with respondent ID as a random effect and the rest of the measured explanatory variables as fixed effects (Supplementary Tables 1–5). We constructed a linear model (LM) or a generalised linear model (GLM), for each of our five main stress scores, according to the distribution of the response variable (see ‘Methods’ section). This analysis revealed that as days passed, and accounting for the effects of other time-varying or demographic variables, people reported significantly lower levels of distress on all of the five stress-related scores, except concern about contracting COVID-19 (Supplementary Tables 1–5; FA1: b = −1.4e−3; *p* = 6.6e−6; FA2: *b* = −2.8e−4; *p* = 0.71; FA3: *b* = −1.9e−3; *p* = 0.016; number of symptoms: *b* = −5.3e−3; *p* = 0.012; number of coping strategies: *b* = −7e−3; *p* = 1.1e−15). Furthermore, we found a significant correlation between the number of responses submitted by each participant and (1) the general emotional distress scale (Supplementary Table 1; *b* = −4.6e−3; *p* = 1.9e−12) and (2) the number of stress-related symptoms experienced (Supplementary Table 4; *b* = −1.7e−2; *p* = 1.5e−3). Finally, this analysis revealed that the number of new daily COVID-19 cases was correlated with all five stress-related scores (Supplementary Tables 1–5; FA1: *b* = 5.1e−4; *p* < 2e−16; FA2: *b* = 1.2e−3; *p* < 2e−16; FA3: *b* = 7.9e−4; *p* = 4.7e−12; symptoms: *b* = 1.8e−3; *p* = 5.1e−7; coping: *b* = 9.4e−4; *p* = 3.7e−9).

Overall, these correlations may demonstrate our questionnaire’s sensitivity in capturing stress-related effects of COVID-19 in real-time. Interestingly, a similar correlation between stress symptoms (assessed by social media data mining) and the number of new COVID-19 cases were found in a study done in the United States [18].

### Women report higher levels of distress than men

**Fig. 2:**
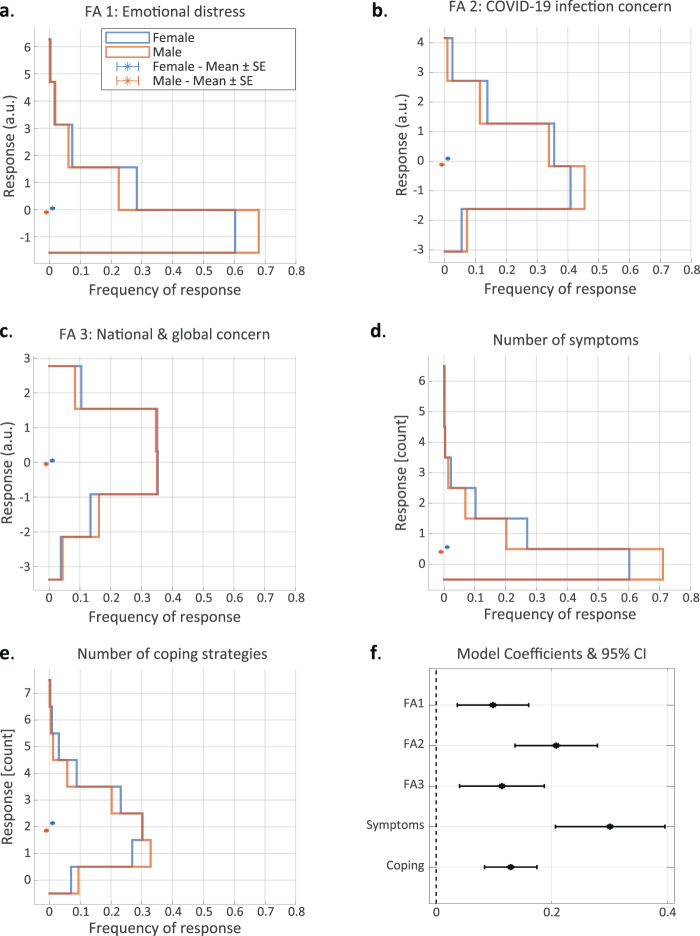
Women report higher levels of distress than men. **a**–**e** Stress-related response distributions of women (blue) and men (orange): **a** general emotional distress score; **b** COVID-19 infection concern scale; **c** national-global concern scale; **d** number of stress-related symptoms; **e** number of stress-coping strategies. **f** 95% confidence intervals for the model coefficients of female vs. male responses on the five stress-related scores. Asterisks in **a**–**e** represent scale means.

Next, we asked whether the emotional response to the pandemic differed between genders (see note regarding non-binary genders in ‘Methods’ section). For these analyses, we only used each participant’s first response, since participants in certain demographic subgroups were more likely to participate more than once (see further explanations in the ‘Methods’ and ‘Discussion’ sections). Similar to previous studies [19, 20], we found that female respondents reported higher levels of distress on the general emotional distress scale (Fig. 2a; Mann–Whitney *U* test; *n*_M_ = 2178, *n*_F_ = 2473; *U* = 0.55; *p* = 1.7e−9); the COVID-19 infection concern scale (Fig. 2b; *n*_M_ = 2178, *n*_F_ = 2473; *U* = 0.55; *p* < 4.5e−9); and the national-global concern scale (Fig. 2c; *n*_M_ = 2178, *n*_F_ = 2473; *U* = 0.52; *p* < 0.024). In accordance with the higher levels of emotional distress, we also found that women reported experiencing a greater number of stress-related symptoms (Fig. 2d; *n*_M_ = 2212, *n*_F_ = 2525; *U* = 0.56; *p* = 2e−14), and using more stress-coping strategies (Fig. 2e; *n*_M_ = 2212, *n*_F_ = 2525; *U* = 0.56; *p* = 4e−13). Specifically, women were more likely to report experiencing increased heart rate (Supplementary Fig. 3; *n*_M_ = 2212, *n*_F_ = 2525; see per-question contingency table counts in figure; Fisher’s exact test: odds ratio (OR) = 1.69; *p* = 0.0026), increased appetite (Supplementary Fig. 3; OR = 1.56; *p* = 0.0006) and trouble sleeping (Supplementary Fig. 3; OR = 1.44; *p* = 7.2e7). Women were also more likely to report using specific coping strategies, such as contacting someone for support (Supplementary Fig. 3; OR = 2.79; *p* < 1e−26) and exercising or meditating (Supplementary Fig. 3; OR = 1.19; *p* = 0.0434). This may suggest that more coping methods are needed to alleviate a greater sense of emotional distress. Importantly, considering the full distribution of responses, the differences between genders seem much more prominent at the lower levels of general emotional distress, as well as for lower numbers of symptoms and coping strategies (Fig. 2a, d–e). In contrast, they seem less prominent for both high and low levels of concern about COVID-19 and about the national and global situation (Fig. 2b, c).

In order to assess whether these gender-related effects were mediated by other measured variables, we constructed an LM or GLM (according to the response distribution; see ‘Methods’ section), for each of the five main stress scores, using only the first response of each participant (henceforth ‘the first-response models’). Essentially, these models quantify the relationship between gender and levels of distress, while accounting for the effects of all the other measured explanatory variables. This analysis revealed that, even with all the other measured variables adjusted for, women reported higher levels of distress on all five main stress scores (Fig. 2f; FA1: *b* = 0.1; *p* = 0.002; FA2: *b* = 0.21; *p* = 1.1e−8; FA3: *b* = 0.11; *p* = 0.002; number of symptoms: *b* = 0.3; *p* = 4.7e−10; number of coping strategies: *b* = 0.13; *p* = 2.4e−8).

### Age negatively correlates with reported distress

**Fig. 3:**
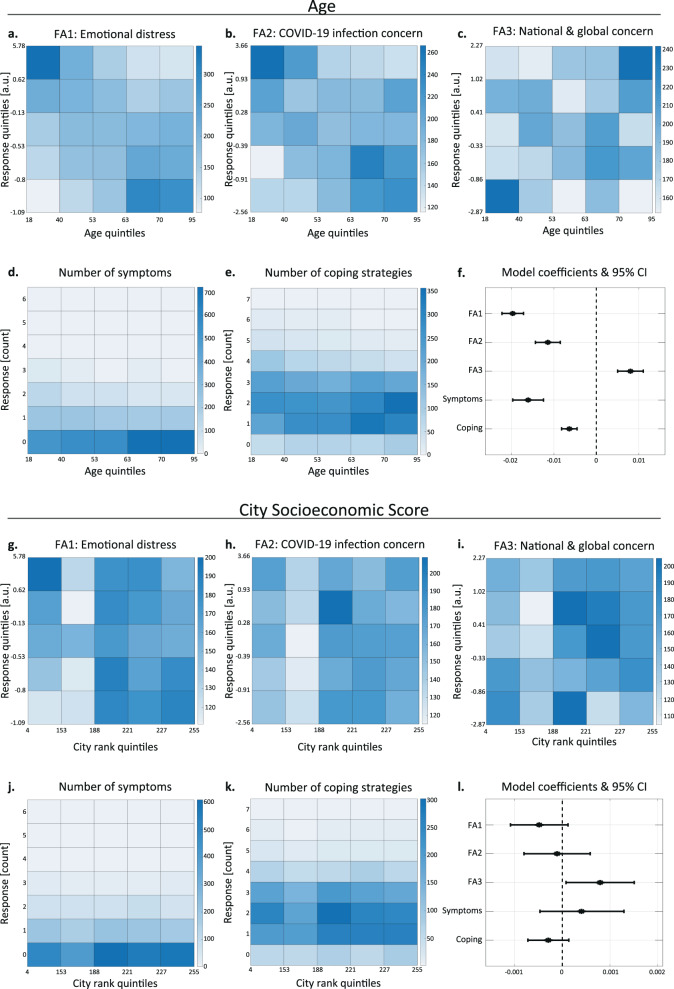
Age and socioeconomic status correlate with reported distress. **a**–**e** Heatmaps representing the frequency of responses for each age-response subgroup. Age and the three continuous response scores (**a**–**c**) were divided into five equally sized subgroups based on quintiles, while the (integer) number of symptoms and coping strategies were left as is (**d**, **e**; see ‘Methods’ section). Kendall’s correlation coefficient with Bonferroni correction for multiple comparisons: **a** *n* = 4641; Tau = −0.2; *p* < 1e−86; **b** *n* = 4641; Tau = −0.11; *p* < 1e−25; **c** *n* = 4641; Tau = 0.05; *p* = 8.38e−6; **d** *n* = 4728; Tau = −0.12; p < 1e−22; **e** *n* = 4728; Tau = −0.09; *p* < 1e−15. **f** Model coefficients and 95% confidence intervals for the association between age and responses on the five stress-related scales. **g**–**k** Heatmaps representing the frequency of responses for each city socioeconomic score (CSS)-response subgroup pair. CSS and the three continuous response scores (**g**–**i**) were divided into five equally sized subgroups based on quintiles, while the (integer) number of symptoms and stress-coping strategies (**j**, **k**) were left as is (see ‘Methods’ section). Kendall’s correlation coefficient with Bonferroni correction for multiple comparisons: **g** *n* = 4009; Tau = −0.041; *p* = 7.9e−4; **h** *n* = 4009; Tau = 0.02; *p* = 0.33; **i** *n* = 4009; Tau = 0.032; *p* = 0.0164; **j** *n* = 4081; Tau = 0.026; *p* = 0.191; **k** *n* = 4081; Tau = −0.033; *p* = 0.026. **l** Model coefficients and 95% confidence intervals for the association between CSS and responses on the five stress-related scales.

Since age is a known factor influencing the stress response [21], we evaluated the effect of age on people’s emotional responses to the pandemic. Since participants in certain age groups were more likely to participate more than once, we only used each participant’s first response for these analyses (see further explanations in the ‘Methods’ and ‘Discussion’ sections). To visualise these potential associations, we divided the respondents into five equally sized subgroups according to their age, and divided the main continuous response scores (for the general emotional distress, COVID-19 infection concern, and national-global concern scales) into five subgroups according to the responses. We then plotted the frequency of each pair of age-response groups (Fig. 3a–e; see ‘Methods’ for details). Under the null hypothesis of no association between respondents’ age and their responses, each matrix cell is expected to have the same frequency, in stark contrast to the matrices we obtained.

We then quantified the correlation between respondents’ age and their answers, and found that younger respondents scored significantly higher on the general emotional distress scale (Fig. 3a; Kendall’s correlation coefficient with Bonferroni correction for multiple comparisons: *n* = 4641; Tau = −0.2; *p* < 1e−86), and on the COVID-19 infection concern scale (Fig. 3b; *n* = 4641; Tau = −0.11; *p* < 1e−25). This reduction in concern with age may seem counterintuitive, in light of the significantly increased risk for complications among older COVID-19 patients [22], but it is consistent with several previous studies [6, 11, 23]. Age was also negatively correlated with the number of stress-related symptoms reported (Fig. 3d; *n* = 4728; Tau = −0.12; *p* < 1e−22) and with the number of stress-coping strategies used (Fig. 3e; *n* = 4728; Tau = −0.09; *p* < 1e−15). Importantly, despite a lower total number of coping strategies, older respondents exhibited an increased tendency to exercise and/or meditate (Supplementary Fig. 4; Mann–Whitney *U* test: *n* = 4728; *U* = 0.58; *p* < 1e−21). Using positive coping strategies, such as exercise, was also reported for the elderly US population [24]. Intriguingly, in contrast with all other types of concern and distress, older respondents scored higher on the national-global concern scale (Fig. 3c; *n* = 4641; Tau = 0.05; *p* = 8.38e−6), supporting the separation of these concerns into discrete factors.

Next, we used the first-response models (see previous section, ‘Discussion’ and ‘Methods’) to assess whether these age-related effects were mediated by other measured variables. This analysis revealed that even with all other measured variables accounted for, very similar to the marginal correlations described above, younger participants still scored significantly lower on the national-global concern scale, and significantly higher on all four remaining stress scores (Fig. 3f; FA1: *b* = −0.02; *p* = 1.2e−50; FA2: *b* = −0.01; *p* = 2.5e−14; FA3: *b* = 8e−3; *p* = 1.8e−7; symptoms: *b* = −0.02; *p* = 4.28e−18; coping strategies: *b* = −6.4e−3; *p* = 8.8e−12).

Taken together, these analyses reveal that the pandemic is affecting younger people’s mental and emotional states more severely.

### High city socioeconomic status is associated with higher national and global concern

Socioeconomic status is a strong predictor of health outcomes, and is generally associated with distress and with the prevalence of mental health problems [25]. To evaluate the association between the respondents’ socioeconomic status and their emotional response to the pandemic, and since we did not have details about the respondents’ income, we used their city socioeconomic score (CSS). The CSS is provided by the Israeli Central Bureau of Statistics (cbs.gov.il), which scores all cities, towns and other incorporated settlements in Israel from 1 to 255, with higher numbers corresponding to a higher status. To visualise this relationship in detail, we divided the respondents into five equally sized subgroups according to their CSS, and the continuous responses (general emotional distress, COVID-19 infection concern and national-global concern scales) into five groups according to extent quintiles, and plotted the frequency of each pair of CSS-response groups (Fig. 3g-k; see ‘Methods’ for details, Supplementary Fig. 5). Under the null hypothesis of no association, we would expect all matrix cells to have a similar frequency. Instead, respondents with low CSS appeared more likely to report higher levels of general emotional distress (Fig. 3g). However, when we accounted for all the other measured explanatory variables, which may mediate or confound this effect, using the first-response models (see previous sections, ‘Discussion’ and ‘Methods’) this apparent association was not statistically significant (Fig. 3l; *b* = −4.8e−4; *p* = 0.116). In contrast, respondents with low CSS seemed more likely to report lower levels of national and global concern (Fig. 3i). This association was statistically significant (Fig. 3l; *b* = 7.9e−4; *p* = 0.03) even when we accounted for all the other measured explanatory variables.

### Employment status is associated with reported distress

As the pandemic influenced the stability of workplaces [26–28], we assessed the association between respondents’ employment status and their emotional response to the pandemic. We divided the respondents into four groups: (1) respondents who are currently working (*n* = 2546); (2) respondents who lost their job due to COVID-19 (either termination of employment (ToE), or on paid or unpaid leave, or forced retirement; *n* = 700); (3) respondents who were unemployed since before the pandemic (*n* = 405); and (4) respondents who were retired since before the pandemic (*n* = 1131).

**Fig. 4:**
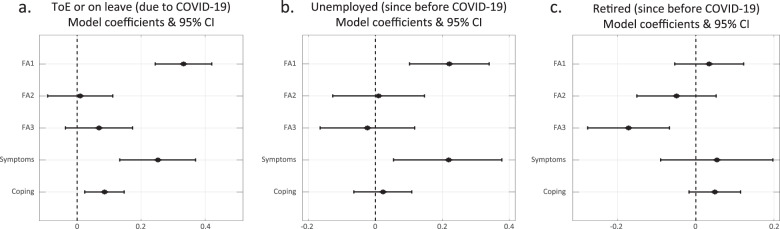
Employment status is associated with reported distress. **a**–**c** Model coefficients and 95% confidence intervals for the association between employment status (relative to currently working respondents) and respondents’ stress-related responses. **a** Respondents who lost their job due to COVID-19 (either termination of employment (ToE)), or on paid or unpaid leave, or forced retirement; **b** respondents who were unemployed since before the pandemic; **c** respondents who were retired since before the pandemic.

We then plotted the response distributions of each of these subgroups (Supplementary Fig. 6) and used the first-response models (with group 1—‘currently working’—as the reference level) to quantify their association, while accounting for the other explanatory variables (Fig. 4a–c).

We found that currently unemployed participants reported significantly higher levels of general emotional distress (unemployed due to the pandemic: Fig. 4a; *b* = 0.33; *p* = 2e−13; unemployed since before the pandemic: Fig. 4b; *b* = 0.22; *p* = 2.8e−4) and a significantly higher number of stress-related symptoms (unemployed due to the pandemic: Fig. 4a; *b* = 0.25; *p* = 3e−5; unemployed since before the pandemic: Fig. 4b; *b* = 0.22; *p* = 0.008), compared to currently employed respondents. Moreover, respondents whose employment was terminated or were on leave due to COVID-19 reported using significantly more stress-coping strategies compared to currently working respondents (Fig. 4a; *b* = 0.09; *p* = 6.4e−3). In contrast, retired respondents reported lower levels of national and global concern (Fig. 4c; *b* = −0.17; *p* = 0.001). Interestingly, despite the negative association between age and emotional distress, with age accounted for, retired participants did not report significantly different levels of emotional distress compared to currently working participants (Fig. 4c; FA1: *b* = 0.03; *p* = 0.448). Taken together, these results may exemplify the roles employment plays in providing both financial security, social support, and better self-esteem [29], which ultimately influence emotional state.

### Respondents with prior medical conditions report elevated general distress and concern about contracting COVID-19

**Fig. 5:**
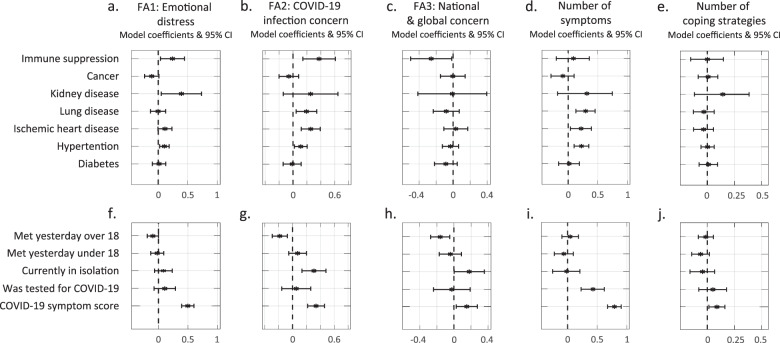
Association between specific medical conditions or risk factors for COVID-19 and stress-related symptoms and coping strategies. Model coefficients and 95% confidence intervals for the association between prior medical conditions (**a**–**e**) and COVID-19-related behaviours and symptoms (**f**–**j**) and respondents’ stress-related responses.

Various medical conditions may increase the risk for complications of COVID-19 [30, 31]. Therefore, we explored whether having prior medical conditions influenced respondents’ emotional distress. We used the first-response models to quantify these associations, while accounting for the other explanatory variables, which may confound or mediate them (Fig. 5a–e). We found that respondents with immune system suppression (ISS), kidney disease (KD) or hypertension reported higher levels of general emotional distress (Fig. 5a; ISS: *b* = 0.24; *p* = 0.02; KD: *b* = 0.39; *p* = 0.024; hypertension: *b* = 0.1; *p* = 0.011). Moreover, respondents with ISS, lung disease (LD), heart disease (HD) or hypertension reported higher levels of concern about contracting COVID-19 (Fig. 5b; ISS: *b* = 0.37; *p* = 0.002; LD: *b* = 0.19; *p* = 0.01; HD: *b* = 0.26; *p* = 2.5e−4; hypertension: *b* = 0.11; *p* = 0.018). Finally, respondents with LD, HD or hypertension reported experiencing more stress-related symptoms (Fig. 5d; LD: *b* = 0.29; *p* = 3.6e−4; HD: *b* = 0.22; *p* = 0.016; hypertension: *b* = 0.22; *p* = 4e−4). In contrast, no medical condition was associated with the number of coping strategies used (Fig. 5e). Similarly, no medical condition was associated with the national and global concern score, except for ISS, which had a significant negative correlation (Fig. 5c; *b* = −0.26; *p* = 0.036).

### COVID-19 symptoms and related behavioural factors associated with reported distress

Next, we examined the extent to which COVID-19-related symptoms and other related environmental factors were associated with reported distress. We used the first-response models to quantify these associations, while accounting for the other explanatory variables, which may confound or mediate them (Fig. 5f–j). First, since social relationships are associated with better physical and mental health [32, 33] and better coping with stressful situations [34], but may also increase the risk of contracting COVID-19, as previously mentioned, we examined whether the number of people respondents had met is associated with their reported distress. Notably, these encounters likely included various types (e.g., meeting family and friends, colleagues, or strangers). We found a nearly significant (*p* = 0.0507) negative correlation between respondents’ general emotional distress and the number of people over the age of 18 with whom they met (Fig. 5f; *b* = −0.09). Furthermore, respondents who met with more adults reported worrying less about themselves or people close to them contracting COVID-19 (Fig. 5g; *b* = −0.18; *p* = 9.8e−4), and reported lower national-global concern (Fig. 5h; *b* = −0.16; *p* = 4.7e−3). In contrast, we found no significant association between the number of people under the age of 18 with whom respondents met and any of the five main scores (Fig. 5f–j).

We further examined the reports of respondents who are potentially at higher risk for contracting COVID-19, namely, being in quarantine, tested for COVID-19, or experiencing COVID-19 related symptoms. Respondents who reported being in quarantine (due to contact with confirmed patients, returning from abroad, having symptoms, or voluntarily isolating oneself) also report significantly higher levels of worrying about contracting COVID-19 (Fig. 5g; *n*_quarantine_ = 181; *n*_not_quarantine_ = 3,964; *b* = 0.31; *p* = 4.6e−4) and of the national-global concern score (Fig. 5h; *b* = 0.18; *p* = 0.047). Furthermore, respondents who reported being tested for COVID-19 reported experiencing more stress-related symptoms (Fig. 5i; *b* = 0.43; *p* = 1.7e−5). Finally, respondents who reported experiencing common symptoms of COVID-19 (see ‘Methods’ for the full list of symptoms and the score used here) also scored significantly higher on all five main scores (Fig. 5f–j; FA1: *b* = 0.5; *p* = 1.6e−20; FA2: *b* = 0.34; *p* = 3.6e−08; FA3: *b* = 0.15; *p* = 0.017; symptoms: *b* = 0.8; *p* = 1.9e−41; coping strategies: *b* = 0.09; *p* = 0.018).

Notably, difficulty breathing may result from both stress and COVID-19 illness, and therefore appears in both questionnaires, which likely contributes to this strong association between both types of symptoms.

## Discussion

This study explored the behavioural, emotional and mental health impact of the COVID-19 pandemic during six weeks, encompassing the end of the first outbreak and the beginning of the second one in Israel. We used clinically validated instruments (BSI-18, PSS, COPE) to assess symptoms and coping strategies, and questions specifically designed to assess COVID-19-related concerns. As expected, in reaction to stressful events, people reported a variety of concerns, mainly related to their close surroundings (their country and relatives). These non-self-centred concerns may reflect an increased sense of belonging to the country/community. Non-self-centred concerns were reported during the initial outbreak in the United States [19] and also during other times of threat in Israel [35].

Importantly, respondents were allowed to answer the survey once a day. On one hand, this enabled us to examine the kinetics of participants’ emotional state over time. On the other, it introduced considerable variation in both the number of responses per respondent and the time intervals between these repeated responses. Most participants (66.8%) responded only once, but a few responded almost every day. Moreover, when we examined the participants who responded more than once, we found they were overall older (59.8 ± 13.9, mean ± STD) and had a higher proportion of women (55.8%) and retired individuals (29.7%) than those who responded only once (age: 53.4 ± 16.3, women: 52%, retired: 20.6%), thus amplifying the slight overrepresentation of these demographic subgroups in our research sample (see ‘Methods’ section). As we have shown, these demographic variables were also associated with levels of reported distress (regardless of whether we used the first or all responses). Therefore, for the distribution plots and analyses that emphasise response differences between different population subgroups, we only used the first responses, which are statistically independent and proportionately represent every subgroup in our research sample. Nevertheless, we found it essential to analyse the dynamics of these repeated responses, along with other time-varying and demographic variables, using statistical models specifically designed for this purpose (Supplementary Tables 1–5). Combining these complementary types of analysis provides the most comprehensive description of the data.

Despite the reported concerns, the anxiety- and depression-related responses (based on the BSI-18 anxiety and depression subscales) are similar to the Israeli norm, based on a nationwide representative sample of 510 community respondents between the ages of 35 and 65 years [15], and are lower compared with Israelis’ scores during war years [36]. This may be due to sampling at a stage at which the pandemic was mostly well-controlled, with a low number of COVID-19 cases and deaths, and without movement restrictions. In the international online survey ECLB-COVID19, which assessed the impact of COVID-19 restrictions, respondents reported a deterioration in mental well-being during home confinement compared to before [37]. Notably, the normal levels of the anxiety and depression subscales of the BSI assessed in a representative Israeli sample were still higher than the normal levels in the USA and UK at the time [15]. In addition, another important point to consider is that our research sample may not accurately represent the general Israeli population: The percentage of respondents with academic education and the average CSS are higher compared to the general population (70% vs. 50.2%, and 187.06 vs. 132.7, respectively [38]).

Even though levels of general emotional distress were similar to the norm, our study describes the inequalities in mental-health burden associated with the COVID-19 pandemic. As shown, a higher emotional burden is associated with being female, younger and unemployed. Our findings add to the reported gender differences that were assessed during the COVID-19 outbreak phase in China and in the United States [4, 19]. It is interesting to compare our findings to those of the UK COVID-19 social study [39, 40]: The number of COVID-19 cases during the initial outbreak was much higher in the UK compared to Israel; therefore, it is not surprising that contracting COVID-19 remains the most prevalent concern in the UK, and was a lesser concern in Israel. However, despite these differences, in both populations being younger and having lower socioeconomic status correlated with increased emotional distress. The level of community resources may influence individuals’ ability to cope with life challenges, and low socioeconomic places of living, characterised by fewer resources, were found to be more vulnerable in Israel [41, 42] in agreement with the conservation of resources theory [43].

Employment instability can have devastating effects on the psychological, economic, and social well-being of individuals and communities [44, 45]. Despite the importance of this topic during crises [46], many open questions remain, especially in the context of the COVID-19 pandemic. In our research sample, the people who lost their job due to the pandemic were similar in their levels of emotional distress to those who were unemployed since before the pandemic (Fig. 3a, b). The utilisation of more coping strategies by the newly unemployed (Fig. 3a) may also reflect higher levels of distress in this group, which are not shared by those who were unemployed before the pandemic.

Throughout our analysis, we observe high accordance between the levels of emotional distress, the number of stress-related symptoms, and the number of stress-coping strategies (Figs. 2a, 4a, g and 5a, b, g). This may suggest that employing multiple stress-coping strategies is a sign of inefficient coping, and/or that using multiple strategies is characteristic of highly emotionally disturbed individuals. It would be interesting to investigate whether assessing the coping strategies people employ could be used to predict their level of distress.

In summary, although we cannot say what is considered a ‘normal’ response to this changing reality, in our research sample the prevalent emotional response to the pandemic was low compared to previous challenging times in Israel. Still, our findings highlight the importance of biological and environmental differences for understanding individuals’ ability to cope with the challenges posed by the pandemic. Such considerations should inform planning and policy for similar events in the future. In light of the dramatic increases in COVID-19 cases in certain parts of the world, and the unprecedented social-economic crisis that Israel and the rest of the world are experiencing, it is of great importance to continue to investigate the long-term mental-health effects of the pandemic and its consequences.

## Methods

### Online survey

This study used a two-stage online questionnaire, the first stage of the questionnaire was previously described in detail [47]. In the second stage, respondents reported on the effects of COVID-19 on their psychological and emotional well-being. For more details, including the full questionnaire, please refer to the Supplementary Methods.

### Research sample

Our online questionnaire was made publicly available to anyone with the URL, which was posted and distributed using social media starting on April 28th. For this study, we used data collected until June 9th 2020. During this period, we collected 12,125 responses from 4933 respondents. The instructions clearly stated that the questionnaire was intended for adult (18 years old or above) respondents only, and the 74 respondents who indicated they were <18 years old were discarded from all of the analyses. For details regarding gender, please refer to the Supplementary Methods.

### Categorising questions using factor analyses

Correlations between individual question responses were quantified using Spearman’s rank correlation coefficient. The resulting correlation matrix was used to compute the factor loadings matrix of a common factor analysis model using the ‘factoran’ Matlab function with the ‘promax’ rotation method. A model of three common factors was chosen using both visual inspection of the factor loading matrices corresponding to a range of models with different numbers of common factors, along with the block diagram of the correlation matrix, and using the ‘nScree’ function of the ‘nFactors’ package in R [48–52].

### Statistical analyses

Statistical analyses were performed using Matlab (v.2019b, MathWorks) and R (v-4.0.2 for the univariate analyses and v-4.0.4 for the fixed-effect models). Mixed-effect models were constructed using the package ‘lmerTest’, v. 3.1. All statistical hypothesis tests are two-tailed. Whenever multiple comparisons were made, Bonferroni’s correction was used. For more details, please refer to the Supplementary Methods.

### COVID-19 symptoms score (SRt)

The COVID-19 symptoms score used here was previously described in detail [53]. Briefly, this score aims to reflect the importance of each symptom with respect to its prevalence in confirmed COVID-19 patients, as previously reported [54]. The symptoms included in the score calculation were: fever (79% of confirmed COVID-19 patients), shortness of breath (3.5%), cough (58%), fatigue (29.3%), muscle pain (3.8%), sore throat (3.2%), headache (6%) and diarrhoea (5.7%).

## Supplementary information

Supplementary Methods

Supplementary Tables

Supplementary Figures

## Data Availability

To protect the data privacy of the study participants, the dataset cannot be made publicly available. Specific data needed for reproducing results is available from the corresponding author upon reasonable request.

## Code availability

Code needed for reproducing results is available from the corresponding author upon reasonable request.
