# Supplementary material for: Stress-related emotional and behavioural impact following the first COVID-19 outbreak peak
Source: Mol Psychiatry. 2021 Aug 4;26(11):6149–58. doi: 10.1038/s41380-021-01219-6 (PMC8335462; doi:10.1038/s41380-021-01219-6)
Supplement: Supplementary file 1 — Supplementary Methods [file 41380_2021_1219_MOESM1_ESM.docx]

# Supplementary Methods

## Online survey

This study used a two-stage online questionnaire, each of which may be considered as a separate one. The first questionnaire was previously described in detail [47]. Briefly, in the first stage, respondents reported on COVID-19-related physiological symptoms (described in the COVID-19 symptoms section) and behaviours (Fig. 5b-g), as well as background demographic (e.g., gender, age and city/town of residence) and medical information (Fig. 5a, Supp. Fig 6). The latest version of this questionnaire can be access using the following URL: [coronaisrael.org](https://coronaisrael.org)

In the second stage, respondents reported on the effects of COVID-19 on their psychological and emotional well-being. These questions were partly based on the anxiety and depression subscales of the brief symptom inventory 18 (BSI-18) and the perceived stress scale (PSS; see full survey description in the methods section and Supp. Fig. 2). Additional questions were designed to assess reasons for concern specifically related to the COVID-19 pandemic, specific stress-related physiological symptoms experienced and stress-coping strategies taken from the brief-COPE questionnaire [16]. Responses were collected in six common languages in Israel (Hebrew, Arabic, English, Russian, French and Spanish), but since very few responses used languages other than Hebrew, only Hebrew responses were analyzed here. The latest version of this questionnaire can be access using the following URL: [forms.gle/4yoXxA3UBuC8R2L68](https://forms.gle/4yoXxA3UBuC8R2L68)

## The questionnaire

**The effects of the Coronavirus on the Israeli public**

By completing the following questionnaire in full and clicking "submit", you agree to forward your answers to the questionnaire to Prof. Alon Chen’s research group at the Weizmann Institute of Science.
The Weizmann Institute of Science is a scientific research institution and therefore the study is not intended for clinical purposes or for clinical diagnosis.
Filling out this questionnaire will allow crosschecking your answers to the two questionnaires in order to promote the goals of both studies, all according to the privacy policy:
[www.weizmann.ac.il/pages/privacy-policy](http://www.weizmann.ac.il/pages/privacy-policy)
and the participant form:
alonchenlab.com/wp-content/uploads/2020/04/CoronaSurvey_Info.pdf

Please note that the integrated information may identify you to some extent.

Answering this questionnaire is not a substitute for counselling, diagnosis or professional treatment. If you need any of those, we suggest contacting:
Sahar - Network Assistance & Listening - sahar.org.il
Hotline for emergency mental help 1201
Hotline for trauma suffers 1800-363-363

---------------------------------------------------------------------

The following sentences refer to your general feelings in the past day. In every statement, please indicate which option most accurately describes your feelings.

I felt irritated:
Not at all Slightly Moderately Largely Very largely

I felt hopeless:
Not at all Slightly Moderately Largely Very largely

I felt tired and restless:
Not at all Slightly Moderately Largely Very largely

I felt scared or anxious:
Not at all Slightly Moderately Largely Very largely

I felt so depressed that nothing could cheer me up:
Not at all Slightly Moderately Largely Very largely

I felt that every task takes so much energy:
Not at all Slightly Moderately Largely Very largely

I felt worthless:
Not at all Slightly Moderately Largely Very largely

I felt lonely:
Not at all Slightly Moderately Largely Very largely

In addition, in the past day, have you experienced the following (check all that apply)?

Increased heart rate

Increased sweating

Trouble sleeping

Loss of appetite

Increased appetite

Difficulty breathing

None of these are true

In every statement, please indicate which option most accurately describes your feelings.

In the past day, to what extent did you feel unable to deal with important things in your life?
Not at all Slightly Moderately Largely Very largely

In the past day, to what extent did you feel confident in dealing with your personal problems?
Not at all Slightly Moderately Largely Very largely

In the past day, to what extent did you feel things are under your control?
Not at all Slightly Moderately Largely Very largely

To what extent, did you feel that you couldn’t cope with challenges facing you?
Not at all Slightly Moderately Largely Very largely

In every statement, please indicate which option most accurately describes your feelings.

I am worried about contracting the coronavirus.
Not at all Slightly Moderately Largely Very largely

I am worried about people close to me contracting the coronavirus.
Not at all Slightly Moderately Largely Very largely

I am worried about my financial situation.
Not at all Slightly Moderately Largely Very largely

I am worried about the situation in Israel.
Not at all Slightly Moderately Largely Very largely

I am worried about the situation around the world.
Not at all Slightly Moderately Largely Very largely

How do the following statements reflect your coping with the situation in the past day (check all that apply)

I tried to accept the situation and learn to live with it.

I used alcohol or cigarettes in order to relax

I used a prescription drug to relax

I drew strength from belief in G-d.

I contacted a family member or friend for support.

I contacted a professional for support.

I searched for information regarding the situation.

I exercised / did yoga/meditation

I drew strength from my pets.

Other

Did you work before the corona pandemic?

I did not work before the corona pandemic.
I worked as a salaried employee.
I worked as a freelancer.
I am retired.
Other

If you worked before the pandemic, what is the status of your employment now?

I am still working
I am on unpaid leave.
I am on paid leave.
I was fired/retired following Covid-19

What is your level of education?

Less than 12 years of study.
High school diploma (Bagrut).
Technical certificate.
Bachelor’s degree and above.

My Gender:

Female
Male
Other

My Age (in years):________

I live in: ________________

Anything else you would like to add? ______________

## Research sample - Note regarding non-binary genders

Respondents were asked to select for their gender either ‘Male’, ‘Female’ or ‘Other’, but since only eight respondents chose ‘Other’, their responses were disregarded in the gender analyses.

| **Gender** |  |
| --- | --- |
| Males (%) | 46.4 |
| Females (%) | 53.41 |
| Other (%) | 0.19 |
| **Age**, mean (SD)(years) | 55.5 (15.7) |
| **Working Status** |  |
| Currently Working (%) | 53.24 |
| Fired/On leave due to COVID-19 (%) | 14.64 |
| Unemployed (since before COVID-19) (%) | 8.47 |
| Pensioner (%) | 23.65 |
| **Education** |  |
| Bachelor's degree and above (%) | 72.08 |
| Technical certificate (%) | 11.67 |
| High school diploma (%) | 8.9 |
| Less than 12 years of study (%) | 7.37 |

## Statistical analyses

Unless explicitly stated otherwise, statistical analyses were done based on each respondent’s first response, to ensure the basic assumption of independent samples, as discussed in the Introduction and Discussion sections. The term “statistically significant” was used when p<0.05, following the common convention, but p-values are always shown rounded by at most 5e-4.

For examining the participants’ first responses, each response variable (FA1-FA3) was fitted with a linear model, accounting for all the measured explanatory variables of interest, without interactions. The explanatory variables include:

1. SRt
2. Condition: diabetes
3. Condition: hypertension
4. Condition: ischemic heart disease
5. Condition: lung disease
6. Condition: kidney disease
7. Condition: cancer
8. Condition: immune system suppression
9. Age
10. Met yesterday with under 18
11. Met yesterday with over 18
12. Was tested for COVID-19
13. Currently in isolation
14. City socioeconomic score
15. Gender
16. Days from survey start
17. Work status: retired (since before COVID-19)
18. Work status: unemployed (since before COVID-19)
19. Work status: ToE or on leave (due to COVID-19)
20. New daily COVID-19 cases.

The variable ‘employment status’ included four subgroups (see Fig. 4), out of which the most common subgroup – ‘currently working’ – was used as the reference level for the model. FA1 was highly right-skewed, and was therefore log-transformed before the analysis. The number of symptoms and number of coping strategies were modeled with a generalized linear model (GLM), assuming a Poisson distribution. Missing data (523 out of 4,933 samples) for CSS were replaced by the median. For examining repeated responses, a mixed-effect model was fit for each response (with all the explanatory variables used for the first-response model, as well as the response number per participant), including ID as a random factor. For the number of symptoms and number of coping strategies, GLM were fit assuming a Poisson distribution.

For the Mann-Whitney statistic, we denote U=Pr[X<Y]+0.5 Pr[X=Y], where X and Y are randomly chosen observations from the two distributions. Thus, U=1 and U=0 represent complete separation of the distributions, while U=0.5 represents complete overlap. The U statistic and its corresponding p-values and confidence intervals were calculated using the ‘wmwTest’ function of the ‘asht’ package in R [49]. Odds ratios and their corresponding p-values and confidence intervals were calculated using the ‘oddsratio’ function of the ‘epitools’ package in R [50] with Fisher’s exact conditional maximum likelihood estimation option. The Kendall rank correlation coefficient (commonly known as Kendall’s Tau) and its corresponding p-values were calculated using the Matlab ‘corr’ function and its corresponding confidence intervals were calculated using bootstrapping with the Matlab ‘bootci’ function and the ‘bias corrected and accelerated percentile’ method [51, 52]. Visualizing the association between two ordinal variables

Since both our independent and dependent ordinal variables (e.g., stress-related responses, age, city socioeconomic status) had numerous repeated values, heatmaps were used instead of scatter plots to visualize their relationships. The data were divided into five equally numerous bins whenever possible (i.e., whenever distinct quintiles existed). Otherwise, wherever no distinct quintiles existed (e.g., for the number of reported stress-related symptoms in Fig. 4e,k), the raw values were used. We chose to use five bins whenever possible to match the number of possible responses in most of our questions. Under the null hypothesis – that the variables are statistically independent – all heatmap cells are expected to have roughly the same frequency (f = n/d, where n = number of responses and d = number of heatmap cells), which makes it very straightforward to examine visually. Importantly, the correlation coefficients and the corresponding p-values and confidence intervals were quantified using the raw data, i.e., without any binning (see ‘Methods - Statistical analyses’).
