# Supplementary material for: Stress-related emotional and behavioural impact following the first COVID-19 outbreak peak
Source: Mol Psychiatry. 2021 Aug 4;26(11):6149–58. doi: 10.1038/s41380-021-01219-6 (PMC8335462; doi:10.1038/s41380-021-01219-6)
Supplement: Supplementary file 2 — Supplementary Tables [file 41380_2021_1219_MOESM2_ESM.docx]

# Supplementary tables

### **Supplementary table 1. Linear model for the general emotional distress score.** Table summarizing model coefficient estimates and corresponding statistics (see *Methods* section).

### **Supplementary table 2. Linear model for concern about COVID-19.** Table summarizing model coefficient estimates and corresponding statistics (see *Methods* section).

### **Supplementary table 3. Linear model for the national and global concern score.** Table summarizing model coefficient estimates and corresponding statistics (see *Methods* section).

### **Supplementary table 4. Generalized linear model for the number of stress-related symptoms experienced.** Table summarizing model coefficient estimates and corresponding statistics (see *Methods* section).

### **Supplementary table 5. Generalized linear model for the number of stress-coping strategies used.** Table summarizing model coefficient estimates and corresponding statistics (see *Methods* section).
