# Supplementary material for: Stress-related emotional and behavioural impact following the first COVID-19 outbreak peak
Source: Mol Psychiatry. 2021 Aug 4;26(11):6149–58. doi: 10.1038/s41380-021-01219-6 (PMC8335462; doi:10.1038/s41380-021-01219-6)

Supplementary figure legends

Supplementary Figure 1. Stress-related response distributions. (a-d, e-h, i-l) Distributions of responses to emotional distress questions among all respondents. Red circles represent response means. (m, n, o) Zoomed-in view of the response means shown in panels a-d (m), e-h (n), i-l (o).

Supplementary Figure 2. Categorizing stress-related questions using factor analysis. (a) Factor loadings of individual participants' first stress-related responses onto the three main factors inferred using factor analysis. (b) Correlation matrix showing the correlation between each pair of questions. The block diagonal structure demonstrates the division into question subsets. To visualize the correlation more clearly, the ordering of the responses of two questions (marked with an asterisk in the figure) was reversed (i.e., 'Not at all' = '5' and 'Very largely' = 1) during this analysis, to match the "valence" of the other questions: "To what extent did you feel confident in dealing with your personal problems" and "To what extent did you feel things are under your control".

Supplementary Figure 3. Association between gender and stress-related symptoms and coping strategies. Left: Table includes the number of positive and negative answers for women and men, as well as the p-values for the association between gender and each stress-related symptom and coping strategy. Right: Odds Ratio and 95% confidence intervals for female *vs*. male responses on each variable.

Supplementary Figure 4. Association between age and stress-related symptoms and coping strategies. Left**:** Table includes the number of positive and negative answers, as well as the p-values for the association between age and each stress-related symptom and coping strategy. Right: Mann-Whitney U and 95% confidence intervals for each variable.

Supplementary Figure 5. Association between city socioeconomic status and stress-related symptoms and coping strategies. Left**:** Table includes the number of positive and negative answers, as well as the p-values for the association between socioeconomic status and each stress-related symptom and coping strategy. Right: Mann-Whitney U statistic and 95% confidence intervals for each variable.

Supplementary Figure 6. Response distributions for each employment status subgroup. (a-e) Left: response distributions and means for each of the four work-status subgroups. Right: Zoomed-in views of the four mean responses are shown for clearer comparison.

Supp. Figure 1

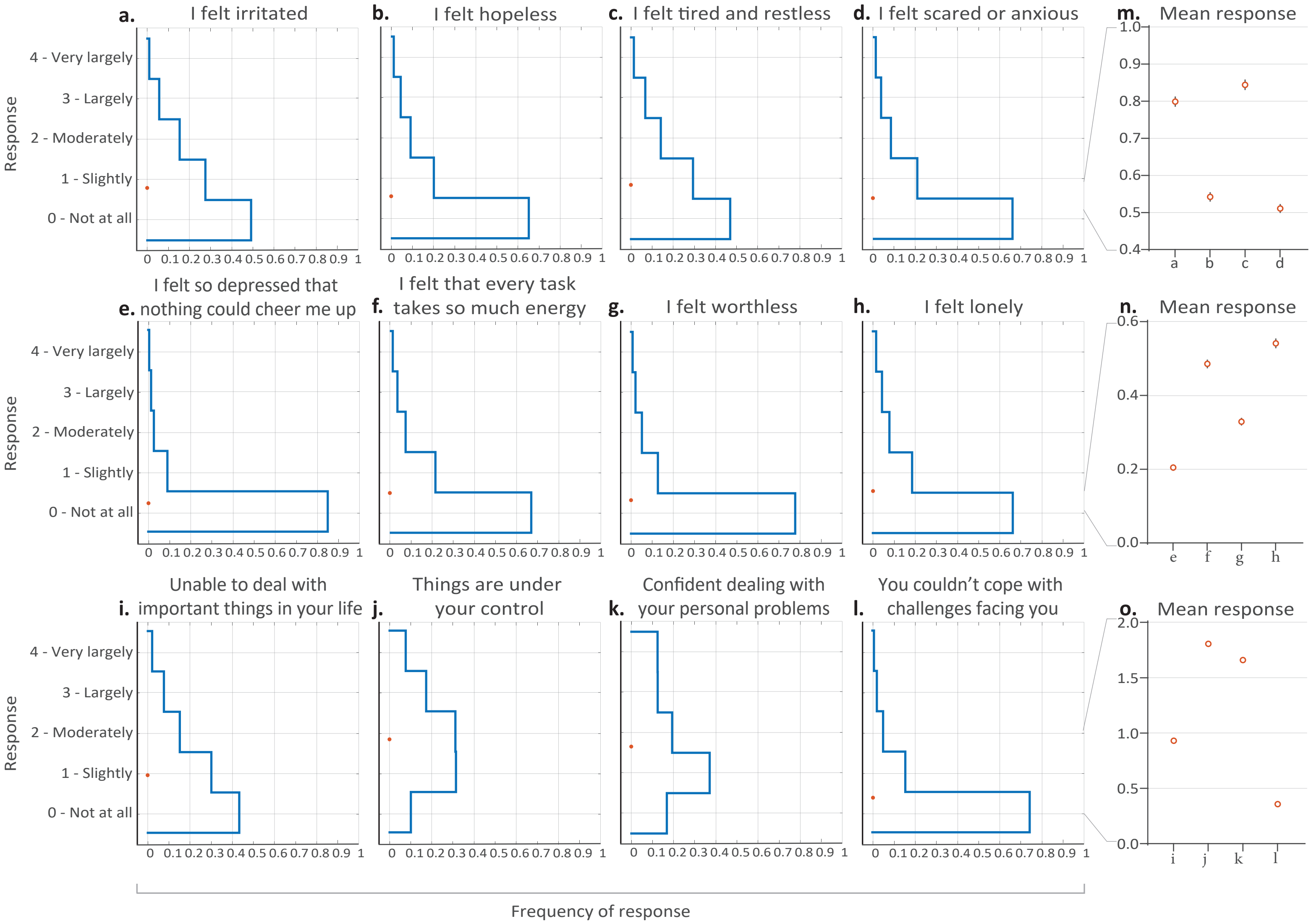

Supp. Figure 2

a.

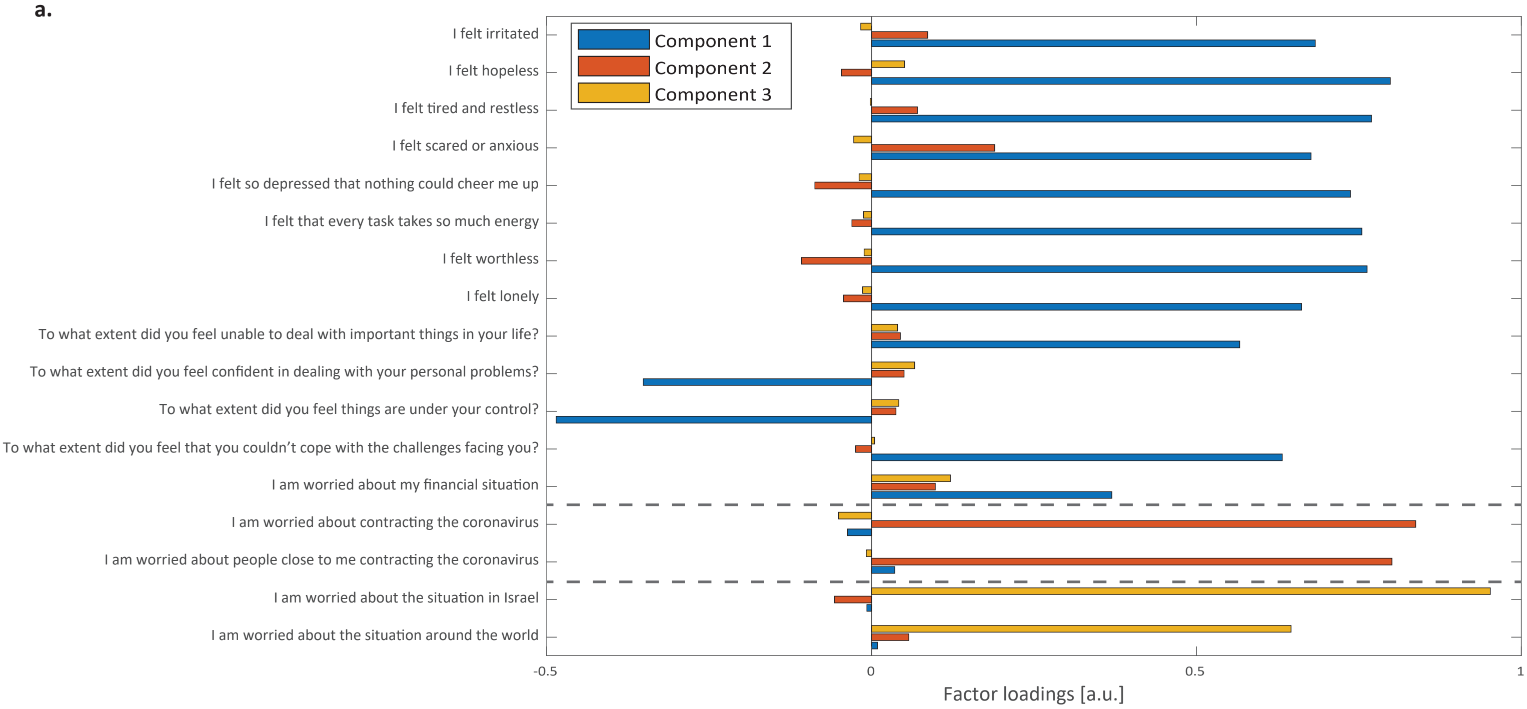

b.

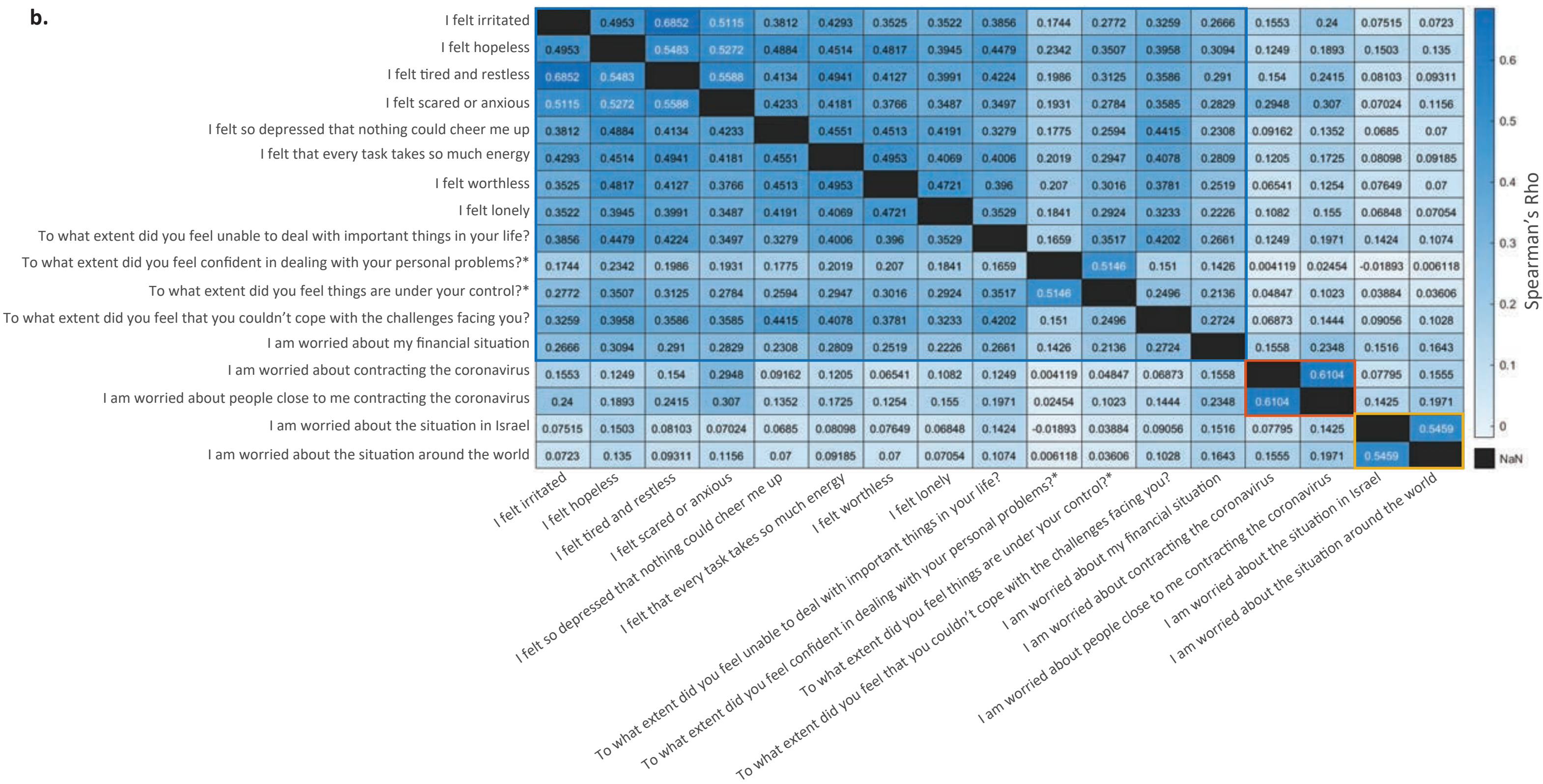

Supp. Figure 3

Did not report		Did report			
F	M	F	M	P-val	
1776	1711	749	501	7.26e-07	Trouble sleeping
2279	2069	246	143	0.00063	Increased appetite
2376	2133	149	79	0.00263	Increased heartrate
2410	2131	115	81	0.86604	Loss of appetite
2434	2155	91	57	0.49598	Difficulty breathing
2455	2174	70	38	0.24794	Increased sweating
907	811	1618	1401	0.99999	I tried to accept the situation and learn to live with it
1252	1193	1273	1019	0.04344	I exercised, did yoga or meditated
1472	1346	1053	866	0.69043	I searched for information regarding the situation
2079	2054	446	158	< 1e-10	I contacted a family member or friend for support
2150	2008	375	204	4.48e-08	I drew strength from my pets
2275	2005	250	207	0.99999	I drew strength from belief in God
2367	2094	158	118	0.95894	I used alcohol or cigarettes in order to relax
2391	2121	134	91	0.57504	I used a prescription drug to relax
2443	2172	82	40	0.02569	I contacted a professional for support

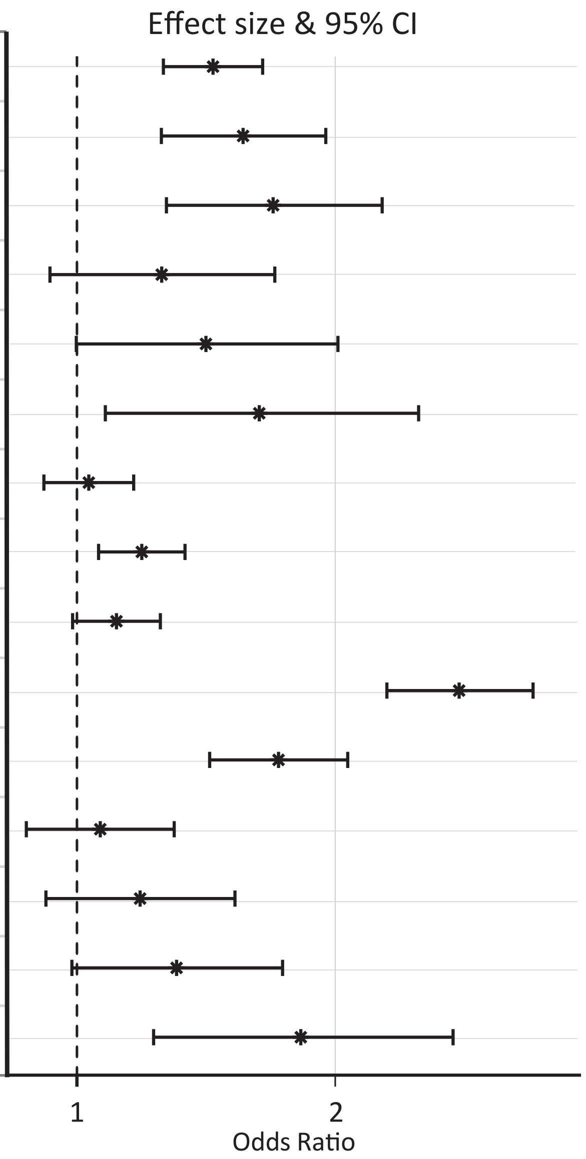

Supp. Figure 4

Did not report	Did report	P-val	
3479	1249	5.71e-08	Trouble sleeping
4338	390	2.52e-06	Increased appetite
4500	228	1.10e-06	Increased heartrate
4532	196	< 1e-10	Loss of appetite
4580	148	0.08536	Difficulty breathing
4620	108	2.68e-05	Increased sweating
1718	3010	0.50062	I tried to accept the situation and learn to live with it
2442	2286	< 1e-10	I exercised, did yoga or meditated
2812	1916	0.13584	I searched for information regarding the situation
4125	603	< 1e-10	I contacted a family member or friend for support
4152	576	5.64e-08	I drew strength from my pets
4272	456	< 1e-10	I drew strength from belief in God
4452	276	< 1e-10	I used alcohol or cigarettes in order to relax
4503	225	> 1-1e-10	I used a prescription drug to relax
4606	122	< 1e-10	I contacted a professional for support

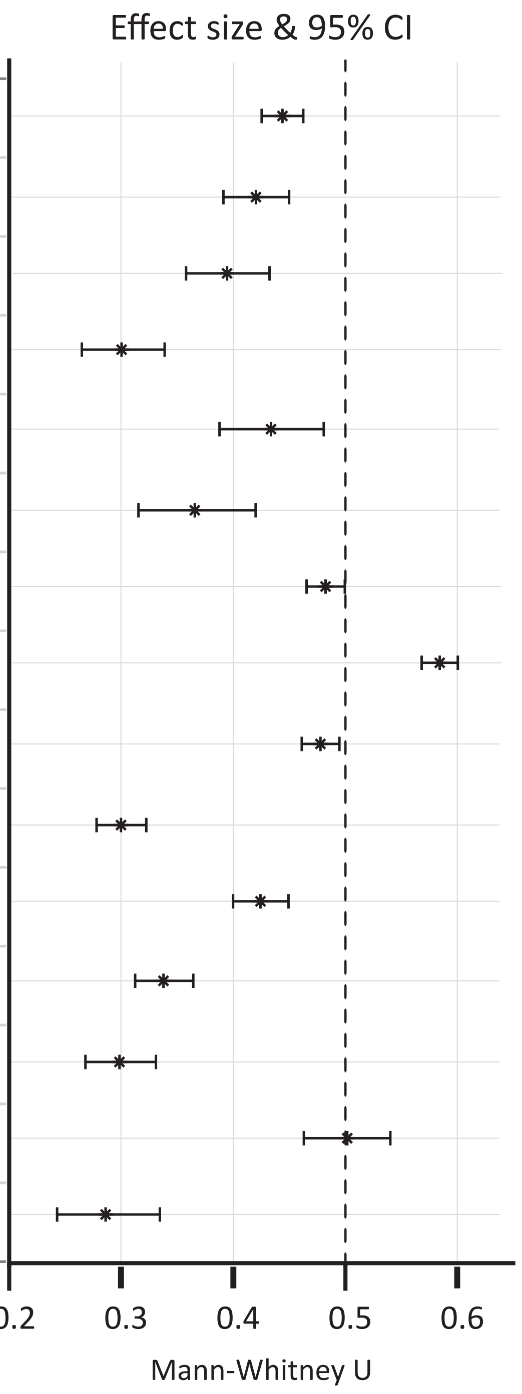

Supp. Figure 5

Did not report	Did report	P-val	
2982	1106	0.92715	Trouble sleeping
3756	332	> 1-1e-10	Increased appetite
3880	208	0.99995	Increased heartrate
3901	187	0.19977	Loss of appetite
3954	134	0.037233	Difficulty breathing
3990	98	> 1-1e-10	Increased sweating
1450	2638	0.9998	I tried to accept the situation and learn to live with it
2130	1958	0.00125	I exercised, did yoga or meditated
2406	1682	0.99992	I searched for information regarding the situation
3553	535	0.00509	I contacted a family member or friend for support
3600	488	0.06136	I drew strength from my pets
3686	402	< 1e-10	I drew strength from belief in God
3830	258	0.97593	I used alcohol or cigarettes in order to relax
3885	203	0.38714	I used a prescription drug to relax
3977	111	> 1-1e-10	I contacted a professional for support

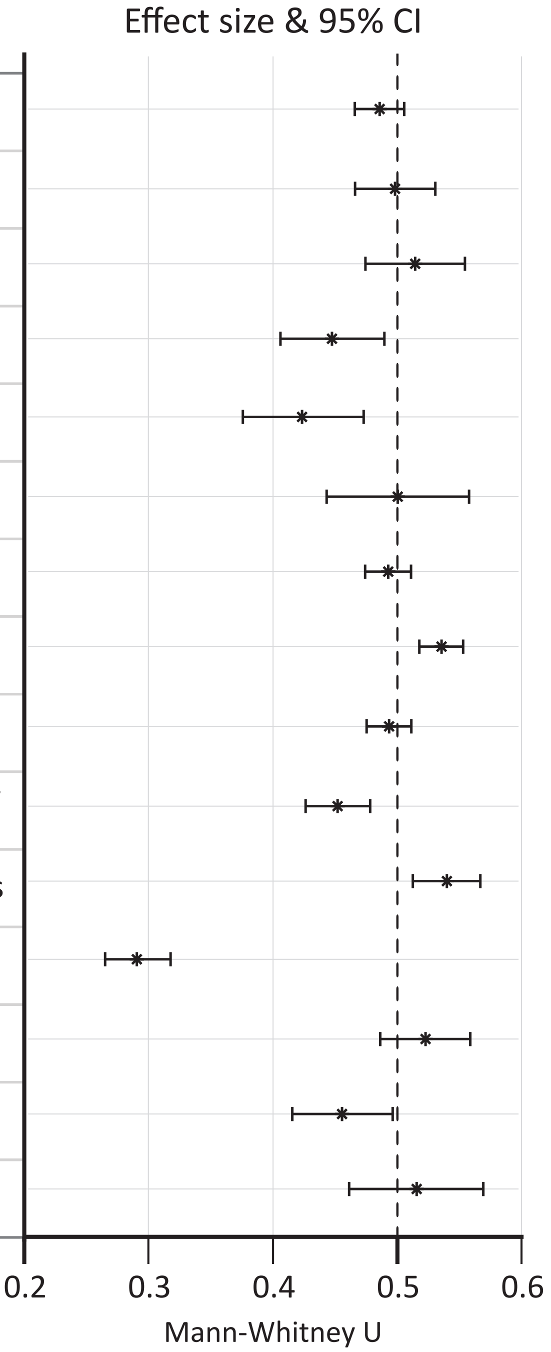

Supp. Figure 6

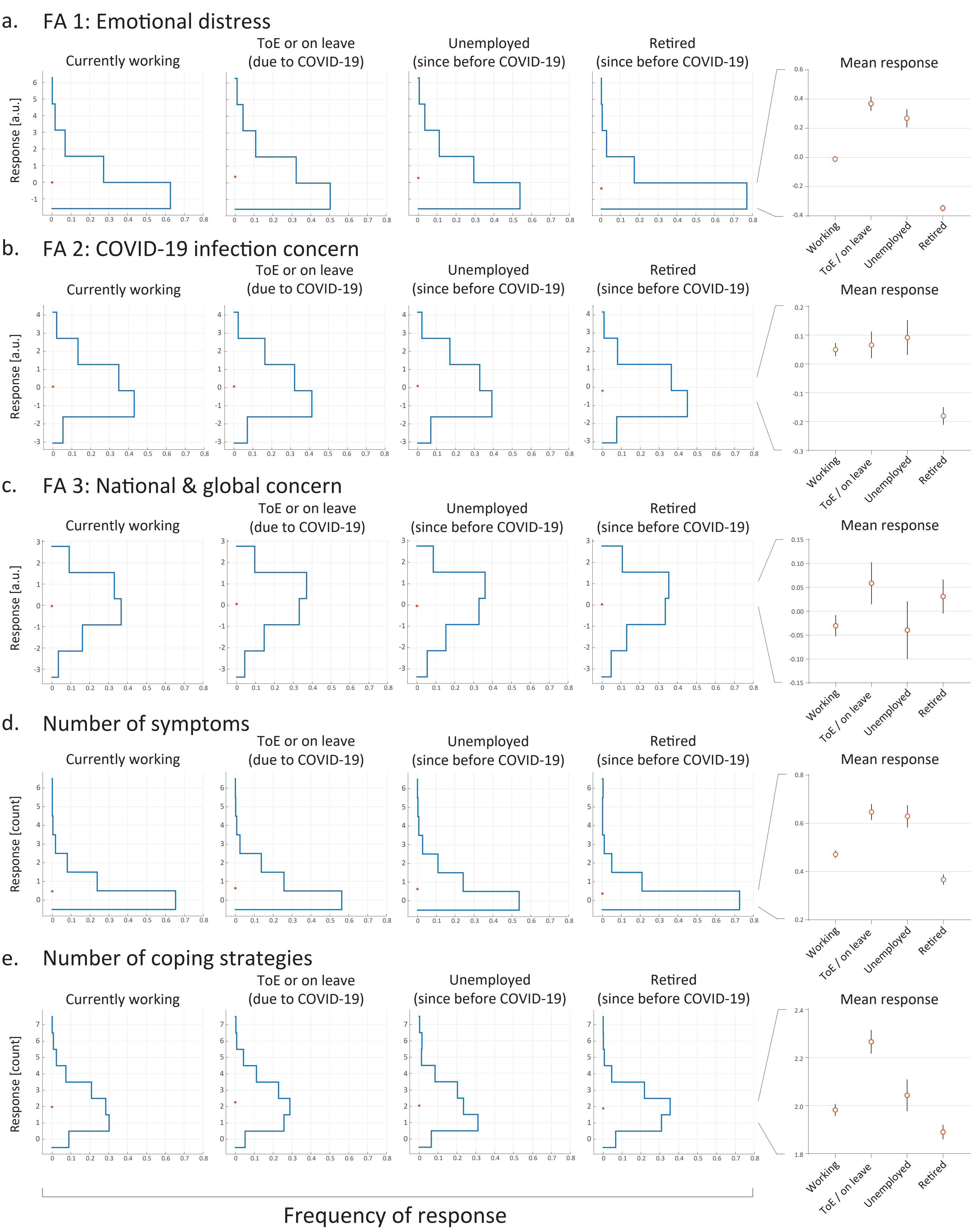

Supplement: Supplementary file 3 — Supplementary Figures [file 41380_2021_1219_MOESM3_ESM.pdf]
